# A Sudden Unilateral Visual Field Loss in a Recreational Tennis Player: Cervical Internal Carotid Artery Dissection Associated With Low-Impact Sports

**DOI:** 10.7759/cureus.59305

**Published:** 2024-04-29

**Authors:** Keijiro Yoshida, Takuma Maeda, Yusuke Nitta, Kaima Suzuki, Hiroki Kurita

**Affiliations:** 1 Cerebrovascular Surgery, Saitama Medical University International Medical Center, Saitama, JPN; 2 Neurosurgery, Tokorozawa Chuo Hospital, Tokorozawa, JPN

**Keywords:** cervical arterial dissection, internal carotid artery dissection, tennis, low-impact sports, antiplatelets, anticoagulants

## Abstract

Traumatic cervical internal carotid artery dissection (CICAD) is a rare condition caused by blunt trauma to the neck, often through automobile- or sports-related collisions, assaults, or falls. Herein, we report an unusual case in which engaging in a low-impact sport (tennis) caused CICAD, without a direct injury. A 56-year-old man with hypertension suddenly experienced a visual field loss in his right eye while playing tennis. Carotid echocardiography revealed severe stenosis of the right internal carotid artery (ICA). Angiography revealed severe and irregular stenosis of the right ICA from the bifurcation to the petrous portion, suggesting CICAD. Upon admission, the patient had left upper visual field defects in his right eye and neck pain. Antiplatelet therapy was initiated with prasugrel (3.75 mg/day), with the intent to treat surgically if the stenosis or symptoms progressed. Follow-up angiography and magnetic resonance imaging showed gradual resolution of the stenosis, and the patient was discharged on day 28 with a modified Rankin Scale score of 1. The CICAD should be considered as a diagnosis for neurological symptoms, even in the context of low-impact sports such as tennis. Antithrombotic therapy is a reasonable first-line treatment for stable CICAD.

## Introduction

Traumatic cervical internal carotid artery dissection (CICAD) is a rare condition caused by direct blunt trauma to the neck, which can arise from various causes such as automobile-related collisions, sports injuries, assaults, or falls [1]. Neck hyperextension, hyperflexion, and head rotation can also lead to CICAD. However, movements during low-impact sports such as tennis have rarely been reported as causes [2-10]. CICAD potentially compromises blood flow to certain areas of the brain and can lead to strokes [1]. Therefore, early recognition and adequate intervention are key to therapeutic success. Herein, we report a rare case of CICAD that occurred without a direct injury, while the patient was playing tennis.

## Case presentation

**Figure 1:**
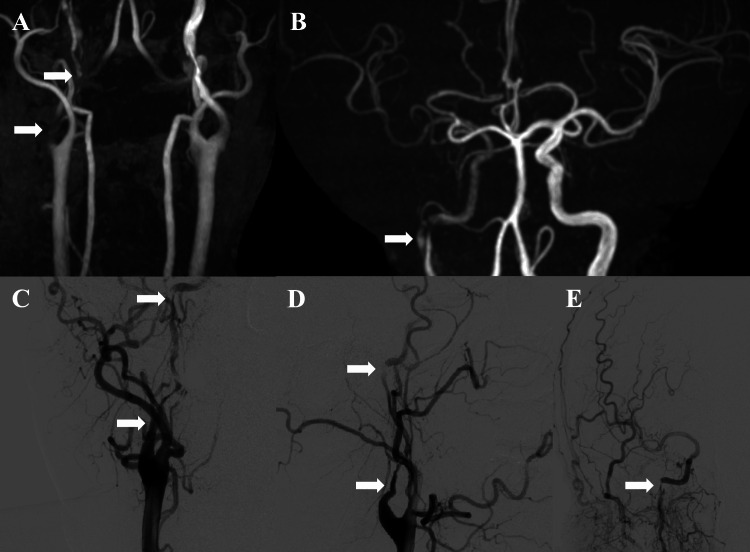
Initial imaging studies. Magnetic resonance angiography (MRA) on day 4 after onset showed poor visualization of the right internal carotid artery (ICA) compared with the left ICA (A and B arrows). A "double lumen" was observed in the petrous portion of the right ICA (B arrow). Digital subtraction angiography on day 5 revealed irregular stenosis from the origin to the petrous portion of the right ICA (C-E arrows).

A 56-year-old man with a history of gastroesophageal reflux disease, asthma, and hypertension experienced sudden visual field loss in the right eye while playing recreational tennis. He had never experienced any similar symptoms before. He was referred to a cardiologist after consulting with an ophthalmologist. The initial ophthalmic examination revealed right nasal hemianopia, and the pre-imaging diagnosis was retinal artery occlusion. However, the fundus examination was almost normal. Carotid echocardiography revealed severe stenosis of the right internal carotid artery (ICA), and the patient was then referred to a neurosurgeon. The initial neurological examination revealed no neurological deficits other than visual field loss. Diffusion-weighted and fluid-attenuated inversion recovery magnetic resonance imaging (MRI) on day 4 after onset showed no apparent abnormalities; however, on magnetic resonance angiography (MRA), the right ICA was poorly visualized compared with the left ICA (Figures 1A-1B). The right carotid angiography performed on day 5 after onset revealed severe and irregular stenosis from the bifurcation of the right ICA to the petrous portion (Figures 1C-1E), and the patient was diagnosed with CICAD. Antiplatelet therapy with prasugrel (3.75 mg/day) was initiated on day 10 after onset, and the patient was transferred to our hospital on day 22 after onset for further evaluation and treatment. 

Upon admission to our hospital, the patient’s level of consciousness was clear. He had a left upper quadrant visual field defect in the right eye and neck pain; however, no other neurological deficits were observed. Limb pulses were palpable. Blood tests showed no hematological abnormalities associated with atherosclerotic risk factors such as diabetes or hyperlipidemia. The initial treatment consisted of medical management with consideration of surgical intervention, including stent placement and high-flow bypass, if the stenosis or symptoms progressed. 

**Figure 2:**
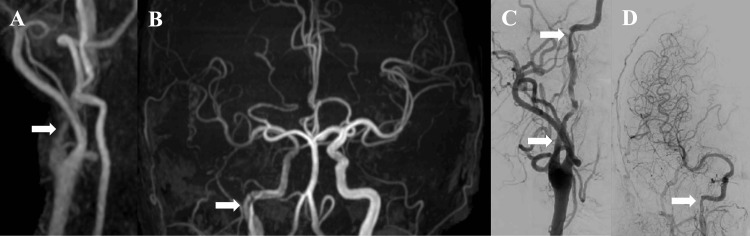
Follow-up imaging studies. Magnetic resonance angiography on day 22 after the onset showed improved visualization of the right internal carotid artery (ICA) (A and B arrows). Right carotid angiography on day 25 after onset showed irregularity of the ICA wall, but partial resolution of the stenosis (C and D arrows).

**Figure 3:**
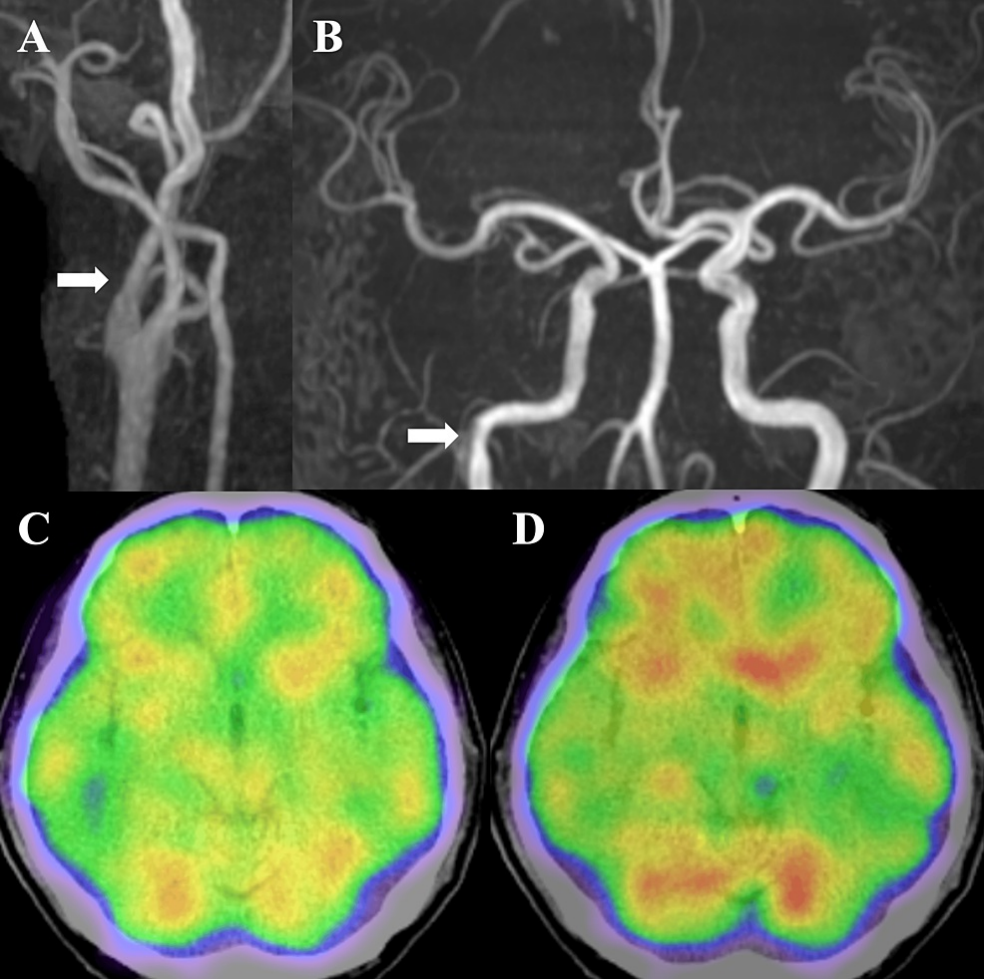
Outpatient follow-up imaging studies. Magnetic resonance angiography performed on day 42 after onset revealed almost no abnormal findings in the right internal carotid artery (A and B arrows). Single-photon emission computed tomography with 123I-isopropyl-p-iodoamphetamine on the same day showed no hypoperfusion area (C: rest and D: acetazolamide stress).

Since the patient had no obvious history of trauma, he was treated according to the guidelines for nontraumatic extracranial arterial dissection. Antiplatelet monotherapy was continued, and the patient was monitored. No symptom progression was observed, and an MRA was performed on day 22 after onset showing improved visualization of the right ICA (Figures 2A-2B). Follow-up angiography of the right common carotid on day 25 after onset revealed irregularity of the ICA wall; however, the stenosis had partially resolved, and the petrous portion of the ICA was clearly visible (Figure 2C). Although cross-flow from the posterior circulation and the contralateral side was observed, the antegrade flow was well preserved (Figure 2D). As the stenosis had resolved, a follow-up MRI was performed without additional treatment. The symptom improved from the right nasal hemianopia to the left upper partial visual field defect in the right eye, and the patient was discharged on day 28 after onset with a modified Rankin Scale score of 1. An MRA was performed on day 42 after onset revealing almost no abnormal findings in the right ICA (Figures 3A-3B). Single-photon emission computed tomography with 123I-isopropyl-p-iodoamphetamine, performed on the same day, showed no hypoperfusion on either side (Figures 3C-3D). The patient is currently being followed up as an outpatient, without any further signs of CICAD progression.

## Discussion

We report a case of CICAD that occurred while the patient was playing tennis, without any obvious traumatic mechanism. The incidence of CICAD is very rare, reported to be 0.08% in patients who have suffered blunt trauma [11]. CICAD is often caused by strong external forces such as automobile collisions and is rarely related to low-impact sports such as tennis [1]. Compared with spontaneous ICA dissection, the susceptible age for traumatic CICAD is younger (approximately 40 years) and the location is more distal (usually around the first to third cervical vertebrae) [12]. The typical symptoms of CICAD include unilateral neck, facial, and head pain, sometimes accompanied by Horner’s syndrome, cerebral infarction, or retinal ischemia [12]. In this case, the fundus examination was normal, and there was no evidence of cerebral infarction. Therefore, the visual field loss may be caused by embolic ischemic optic neuropathy. A diagnosis of dissection can be made by cerebral angiography or MRI/MRA [13]. Basi-parallel anatomical scanning (BPAS) and vessel wall imaging, such as Gd-enhanced T1-weighted sampling perfection with application-optimized contrasts using different flip angle evolution (GdT1-SPACE), are also useful in its diagnosis [14]. 

There have been few reports of arterial dissection in tennis players over the past several decades (Table 1) [2-10]. The age range of patients was 27-63 years. The most common symptoms were neck pain and headache. The most common location was the vertebral artery, with ICA involvement observed in only two cases, including ours. Antithrombotic therapy with antiplatelet or anticoagulant drugs was typically selected for treatment, and only one patient underwent surgery. The clinical outcomes were generally positive. Abe et al. suggested that minor traumas during tennis serve and head swings, involving neck extension and flexion movements, may cause dissection of both the ICA and middle cerebral artery (MCA) [8]. Aljishi and Jayathissa postulated that activities involving hyperextension and rotation of the neck, such as yoga, ceiling painting, anesthesia, and tennis serving, could cause vertebral artery dissection in addition to major trauma [10]. In the present case, the patient had no obvious trauma or history of cervical atherosclerosis. We, therefore, concluded that the dissection was caused by neck extension while the patient was playing tennis. 

**Table 1:** Previous cases of arterial dissection in tennis players. ICA: internal carotid artery; MCA: middle cerebral artery; STA: superficial temporal artery; VA: vertebral artery

Author and year	Age (yrs)	Sex	Symptoms	Location	Treatment	Outcome
Hilton-Jones and Warlow, 1985 [2]	40	M	Left rear neck pain,	VA	N/A	N/A
Roualdes et al., 1985 [3]	N/A	N/A	N/A	VA	N/A	N/A
Giroud et al., 1987 [4]	63	M	Headache, aphasia	ICA	Antiplatelet	Incomplete recovery
Leys et al., 1987 [5]	35	M	Vertigo, diplopia, right limb weakness, neck pain	VA	Anticoagulant	Incomplete recovery
McCrory, 2000 [6]	48	M	Right temporal pain	VA	Anticoagulant	Full recovery
Gabriel et al., 2008 [7]	N/A	N/A	N/A	VA	N/A	N/A
Abe et al., 2009 [8]	27	M	Headache	MCA	STA-MCA bypass	Full recovery
Fragoso et al., 2016 [9]	45	F	Dysarthria, dysmetria, nystagmus	VA	Anticoagulant	Full recovery
Aljishi and Jayathissa, 2018 [10]	38	F	Headache, neck pain, unsteady gait, dizziness	VA	Antiplatelet	Full recovery
Present case	56	M	Visual field loss, neck pain	ICA	Antiplatelet	Incomplete recovery

ICA dissection due to an elongated styloid process, such as in Eagle’s syndrome, has been reported [15]. We considered the possibility of Eagle’s syndrome in our case, but there was no abnormal elongation of the styloid process. 

Antithrombotic therapy using antiplatelet or anticoagulant drugs is the first-line treatment for arterial dissection and should be continued for three to six months [16]. The optimal drug treatment is unknown due to the scarcity of large-scale randomized trials comparing the two treatments. The Cervical Artery Dissection in Stroke Study (CADISS) is the only randomized trial to compare antiplatelet and anticoagulant therapy in patients with extracranial carotid and vertebral artery dissection [17]. Its results showed that there was no difference in ischemic stroke (antiplatelet therapy: 3.2%; anticoagulant therapy: 1.6%), major bleeding (antiplatelet therapy: 0%; anticoagulant therapy: 0.8%), outcomes, or mortality among patients who received either antiplatelet therapy (aspirin, dipyridamole, or clopidogrel, alone or in combination) or anticoagulant therapy (warfarin or heparin, alone or in combination) during one-year follow-up [17]. 

A study reported that the proportion of patients with good prognoses did not differ between those who did or did not receive thrombolytic therapy for CICAD [18]. Moreover, the rates of symptomatic intracranial hemorrhage, mortality, and recurrent stroke were also similar. Further studies are warranted to determine the safety and efficacy of thrombolytic therapy in patients with CICAD.

Surgical treatment should be considered in cases of large vessel occlusion caused by CICAD-associated artery-to-artery embolisms, treatment-resistant progressive or recurrent ischemic lesions, or enlarged pseudoaneurysms. Surgical treatments aimed at restoring blood flow, such as ICA ligation and superficial temporal artery-MCA or high-flow bypass, have been attempted as radical treatments of the embolic source associated with ICA dissection [19]. Recently, stenting has been recommended from the perspective of invasiveness. Emergency stenting stabilizes the intimal flap and prevents deterioration caused by embolization [20]. It is recommended that patients refrain from sports for at least one month [1]. This may be extended if clinical symptoms are not alleviated and imaging findings do not improve.

## Conclusions

CICAD should be considered as a diagnosis for neurological symptoms or neck pain, even in cases related to low-impact sports such as tennis. Antithrombotic therapy represents a reasonable first-line treatment for CICAD. Surgical treatments, including stenting and bypass surgery, should be considered in patients with progressive or recurrent lesions that are refractory to medical therapy.
